# A Novel Polysaccharide from *Sargassum weizhouense*: Extraction Optimization, Structural Characterization, Antiviral and Antioxidant Effects

**DOI:** 10.3390/antiox12101832

**Published:** 2023-10-06

**Authors:** Yi Zhao, Jiaji Chen, Yiqu Ding, Mengyuan Luo, Yanmei Tong, Tingjun Hu, Yingyi Wei

**Affiliations:** 1 Guangxi Key Laboratory of Animal Breeding, Disease Control and Prevention, College of Animal Science and Technology, Guangxi University, Nanning 530005, China; zhaoyi@st.gxu.edu.cn (Y.Z.); chenjiaji@st.gxu.edu.cn (J.C.); dingyiqu@st.gxu.edu.cn (Y.D.); 1818393025@st.gxu.edu.cn (M.L.); yanmeitong@st.gxu.edu.cn (Y.T.); 2 Guangxi Zhuang Autonomous Region Engineering Research Center of Veterinary Biologics, Nanning 530004, China

**Keywords:** *Sargassum* polysaccharide (SP), PRRSV, antioxidant capacity, antiviral effect

## Abstract

Porcine reproductive and respiratory syndrome virus (PRRSV) is one of the most economically important pathogens in the global swine industry over the past three decades. There is no licensed antiviral medication that can effectively control this infection. In the present study, the structure of SP-1 isolated and purified from *Sargassum weizhouense* was analyzed, and its antioxidant capacity and antiviral effect in MARC-145 cells against PRRSV were investigated. The results showed that SP-1 is a novel polysaccharide which mainly is composed of →4)-β-D-ManpA-(1→, →4)-α-L-GulpA-(1→ and a small amount of →4)-β-D-GalpA-(1→. PRRSV adsorption, replication, and release were all suppressed by SP-1. SP-1 therapy down-regulated mRNA expression of the CD163 receptor while increasing the antioxidant gene expression of Nrf2, TXNIP, and HO-1; increasing the protein expression of NQO1 and HO-1; and drastically reducing the protein expression of p-p65. The findings indicated that SP-1 reduces PRRSV adsorption, replication, and release through blocking the expression of the crucial CD163 receptor during infection. Meanwhile, SP-1 exerts antioxidant effects in PRRSV-infected cells through the activation of the Nrf2-HO1 signaling pathway.

## 1. Introduction

Porcine reproductive and respiratory syndrome (PRRS) is an acute and highly contagious viral infectious disease with reportedly high morbidity and mortality [1]. PRRS is currently the most prevalent viral disease endangering pig production in China and is caused by the porcine reproductive and respiratory syndrome virus (PRRSV) [2]. It could lead to miscarriage, preterm birth, stillbirth, sow dyspnea, and subcutaneous purpura, and it can be communicated through a variety of routes, including oral, nasal, respiratory, intraperitoneal, and vaginal [1,3]. Pigs are the only known natural hosts of this infection. PRRSV specializes in porcine alveolar macrophages and causes immunosuppression in pigs. Additionally, African green monkey kidney cell line MARC-145 supports PRRSV infection and proliferation and is commonly used for in vitro virus studies [4]. PRRSV is a single-strand positive-stranded RNA virus with a capsule membrane. Its virions are spherical or oval, the genome is about 15 kb, and the ORF 2–7 gene encodes eight structural proteins (GP2a, GP2b, GP3, GP4, GP5a, GP5, matrix (M), and nucleocapsid protein (N)) [5]. When viruses infect cells, the poison particle must become adsorbed to the cell’s surface and enter the cell, where it dehulls to release the viral DNA. These PRRSV proteins are involved in the process of virus entry into cells, replication, and the assembly of viral particles [6]. The CD163 molecule is a type I membrane protein, also known as the MI130 antigen, and CD163 protein expression is restricted to monocyte/macrophage cell lines. It is the core receptor involved in PRRSV infection and plays a pivotal role in the viral capsid and genomic release [7,8]. Previous research showed that the deletion of CD163 is sufficient to resist PRRSV infection [9]. Therefore, blocking the key viral receptors may preclude the occurrence of viral diseases to a certain extent.

Oxidative stress can be defined as the imbalance between oxidation and antioxidation in the body, and inflammation is a defensive response made by the body to protect itself against oxidative stress [10]. Studies have shown that a PRRSV infection might cause the host to experience oxidative stress and inflammatory reactions [11,12], which interact with and endanger the body’s health. According to Stukelj et al. [13], PRRSV-infected pigs reduced oxidative damage through increasing the levels of antioxidant enzymes such as glutathione peroxidase (GPx) and superoxide dismutase (SOD). Lee et al. [14] observed that anti-PRRSV drugs inhibited virus proliferation through activating inflammatory signaling pathways such as NF-κB and Nrf2-HO1, suggesting that exploring oxidative stress and inflammatory response is the key to the treatment of PRRSV infection. Due to the variability and immune evasion of PRRSV, the traditional control strategies have proven insufficient to provide sustainable and effective protection for pigs, resulting in a state of persistent virus infection in the pig herd. Therefore, the need for a green, safe, and efficient anti-PRRSV infection therapy is urgent.

*Sargassum weizhouense* (*Phaeophyta Sargassum*), native to temperate tropical waters, is rich in proteins, fats, vitamins, and other components. It is used as an ingredient to promote the catabolism of fat and carbohydrates and enhance the body’s immunity in traditional Chinese food. Seaweed’s bioactive components have been widely investigated in recent years [15]. In addition to being full of nutrients, *Sargassum* also has a number of active ingredients such polysaccharides, polyphenols, and terpenes. Among them, polysaccharide, as one of the main chemical components of *Sargassum*, exhibits antiviral effects such as immune regulation and antioxidant and anti-inflammatory activities [16,17]. The *Sargassum* used in this study is one of the characteristic species of *Sargassum*. It is produced near Weizhou Island, Beihai City, Guangxi Zhuang Autonomous Region. A *Sargassum* polysaccharide we isolated previously showed a good anti-inflammatory effect against PCV2 infection in vitro [16], but the structure of the water-soluble polysaccharide was not yet clear.

In the current study, the extraction process of *Sargassum* polysaccharide was improved. The aim of this research was to characterize the structural features of the water-soluble polysaccharide of *Sargassum weizhouense*. Then, the antiviral and regulatory effects of SP-1 on the levels of inflammation and oxidative stress induced by PRRSV infection were assessed in vitro. Our findings can help identify new cellular targets for anti-PRRSV therapy, guide the use of SP-1, and establish a research foundation for the clinical application of antiviral drugs.

## 2. Materials and Methods

### 2.1. Main Chemical Reagents

All reagents are commercially sourced. Sources for basic reagents are provided in the supplemental information. Solarbio Technology Co., Ltd. provided the DEAE-Sepharose Fast Flow (Beijing, China). Sephacryl S200HR was purchased from Sigma-Aldrich Company (Darmstadt, Germany). CCK-8 Cell Counting Kit was purchased from Vazyme Biotech Co., Ltd. (Nanjing, China). GenStar Biosolutions Co., Ltd. (Beijing, China) provided 2× Real Star Fast SYBR qPCR Mix kit. Applied Biological Materials Inc (British Columbia, VIC, Canada) provided All-In-One 5× RT MasterMix. Cell Signaling Technology (Boston, MA, USA) provided the rabbit mAbs for β-actin, phospho-NF-κB p65, HO-1, NQO1, and anti-rabbit IgG HRP-linked antibody. PRRS virus nucleocapsid protein mAb was purchased from GeneTex (SAN Antonio, TX, USA). Millipore (Waltham, MA, USA) provided the chemiluminescent HRT substrate. Both the DAPI staining solution and FITC-labeled goat anti-rabbit IgG (H+L) were bought from Beyotime Biotechnology Co., Ltd. in Shanghai, China.

### 2.2. Plant Materials

*Sargassum* is produced near Weizhou Island, Beihai City, Guangxi Zhuang Autonomous Region. It belongs to the characteristic species of *Sargassum*. This particular *Sargassum* species was officially registered as *Sargassum weizhouense* C.K. Tseng and Lu Baoren in 2002 in the World Register of Marine Species (Aphia ID: 388001).

### 2.3. Isolation and Purification of Sargassum Polysaccharides

As depicted in Figure S1, *Sargassum* powder was treated with 95% ethanol reflux to remove pigments, small molecules, and fat-soluble substances. After adding distilled water and papain (65 °C water bath for 4 h), the supernatant was obtained, and the process was repeated once. The supernatant was collected and concentrated both times, and then it was treated with 2% trichloroacetic acid for 2 h. The sediment obtained after precipitating the supernatant with alcohol was dissolved in distilled water and subsequently freeze-dried to obtain crude *Sargassum* polysaccharides. Crude *Sargassum* polysaccharides were eluted with distilled water or NaCl solution to concentrations of 0.5, 1.0, 1.5, and 2.0 mol/L using DEAE-Sepharose Fast Flow to obtain the components, including SP-1, SP-2, SP-3, SP-4, and SP-5. Absorbance values were measured using the anthrone–sulfuric acid method at 620 nm on a visible spectrophotometer for the eluted fractions. Subsequently, SP-1 was further purified with Sephacryl S200HR. The elution curve was obtained using the anthrone–sulfuric acid method.

### 2.4. Chemical Analysis and Microstructure of SP-1

#### 2.4.1. UV-Vis

The UV-Vis spectrum is recorded through scanning the sample solution (1 mg/mL) at 200–700 nm to determine the presence or absence of protein and nucleic acid contamination of polysaccharides on an ultraviolet spectrophotometer (UV-1750, Shimadzu (Shanghai) Global Laboratory Consumables Co., Ltd., Shanghai, China).

#### 2.4.2. Determination of Molecular Weight (MW)

After SP-1 was purified using Sephacryl S200HR, the MW distribution of SP-1 was determined using the HPGPC technique. The conditions were as follows: liquid-phase systems—U3000 (Thermo Fisher Scientific Inc., Waltham, MA, USA); gel exclusion column—Ohpak SB-805/804/803 HQ (300 × 8 mm); injection volume—20 μL; column temperature—45 °C; flow rate—0.4 mL/min; mobile phase—0.02% NaN_3_, 0.1 M NaNO_3_; elution gradient—isocratic 100 min; differential detector—Optilab T-Rex (Wyatt Technology Corporation, Santa Barbara, CA, USA).

#### 2.4.3. Monosaccharide Composition Analysis

An ion chromatograph (IC, Thermo Fisher Scientific Inc., Waltham, MA, USA) was used for monosaccharide composition analysis. Thirteen kinds of monosaccharide standards (fucose, rhamnose, arabinose, galactose, glucose, and others) were precisely configured with 5 mg/L. Then, 10 mL of TFA (3 M) and 10 mg of SP-1 samples were hydrolyzed at 120 °C for 3 h. After adding 5 mL of water and blow-drying and vortex-mixing the acid hydrolysis solution, precisely aspirating it into the tube nitrogen, it was then centrifuged at 12,000× *g* rpm for 5 min. Ion chromatography was used to analyze the supernatant.

#### 2.4.4. Fourier Transform–Infrared Spectroscopy (FT-IR)

SP-1 was scanned via FT-IR (Nicolet Iz-10, Thermo Scientific, New York, NY, USA) with a scanning range of 4000~400 cm^−1^ after mixing with KBr and cutting into 1 mm slices.

#### 2.4.5. Methylation Analysis

The methylation analysis of SP-1 was performed following Zhu’s method [18]. The primary ion fragments of MS and the peak sequence in GC were examined to determine the glycosidic bond ligation mechanism, which was then used to investigate the derivatives. The following were the mass spectrometry conditions: A full-scan electron bombardment ion source (EI) with a mass scanning range (*m*/*z*) of 50–350 was used to identify SP-1 (Agilent 5977B; Agilent Technologies, Santa Clara, California, USA). The injection volume was 1 μL, and the gas chromatography conditions were as follows: 140 °C for 2.0 min, 3 °C/min to 230 °C, acting for 3 min (Agilent 7890A; Agilent Technologies, Santa Clara, CA, USA).

#### 2.4.6. NMR Analysis

Fifty milligrams of SP-1 was fully dissolved into deuterium oxide (D_2_O) and configured into a 50 mg/mL polysaccharide solution for NMR spectroscopy. 1D-NMR (^1^H-NMR, ^13^C-NMR) and 2D-NMR (COSY, HSQC, HMBC, NOESY spectra) were recorded at 25 °C with a 500 MHz NMR spectrometer (Bruker, Rheinstetten, Germany).

#### 2.4.7. Scanning Electron Microscopy (SEM)

The morphology of SP-1 was observed via a ZERSS MERLIN compact at an acceleration voltage of 5 kV (Carl Zeiss AG, Jena, Germany). In short, approximately 10 μg of SP-1 powder was mounted on the adhesive tape with electrical conductivity. At a working distance of 11.5 mm, the typical characteristics were visualized at magnification factors of 500× and 2000×.

#### 2.4.8. X-ray Diffraction (XRD) Detection

The crystalline structure of SP-1 was evaluated via XRD analysis. Twenty milligrams of SP-1 was weighed on the stage, pressed and spread evenly, and scanned on the X’ Pert Pro X-ray diffractometer (PANalytical B.V., Almelo, Netherlands) in the range from 5 to 80 degrees.

### 2.5. Cells and Viruses

The MARC-145 cell line, purchased from the Institute of Cell Research, Shanghai Institute for Biological Sciences, Chinese Academy of Sciences, was passed down from generation to generation in a 37 °C, 5% CO_2_ condition and frozen by the Basic Veterinary Medicine Laboratory of the College of Veterinary Technology of Guangxi University.

The virus titer of the PRRSV-GXNN1396 strain (Gen bank ID: MD660067) was calculated as 10^−7.33^/0.1 mL. The MOI of PRRSV used in the experiment was 0.01. JM109 strains were preserved and donated by the Preventive Veterinary Medicine Laboratory of Guangxi University.

### 2.6. Cell Viability Assay

MARC-145 cells isolated using trypsin were seeded into 96-well plates at a density of 1 × 10^5^/mL per well with 2% MEM. SP-1 was diluted with 2% MEM to a final concentration of 0, 25, 50, 100, 200, 400, and 800 μg/mL. An SP-1 treatment group (0, 25, 50, 100, 200, 400, and 800 μg/mL), a CCK-8 control group, and a ribavirin group with a concentration of 100 μg/mL were set up. After 24 h or 48 h post treatment, the cell supernatant was discarded, and the premixed CCK-8 dilution, 100 μL/well, was added to the culture for 2 h. The absorbance was detected at 450 nm using a microplate reader. The viability of cells treated with SP-1 was calculated based on the cell control group percentage.

### 2.7. Construction of Standard Quality Granules and Establishment of Standard Plasmid

Total RNA was extracted using RNAiso Plus kits and then transcribed to obtain RNA. Then, the cDNA was obtained using All-In-One 5× RT MasterMix. The gene sequence of the PRRSV N gene was generated via PCR amplification of cDNA with the forward primer 5′-GCCAAATAACAACGGCAAGC-3′ and the reverse primer 5′-GGTTTTTCTTTTCTTTCTTCCCC-3′. After obtaining the amplification product, the sequence was verified through comparing it to the NCBI database. Then, it was restriction-digested and cloned into the pMD-18T vector to form pMD-18T-PRRSV-N. Then, the concentration was measured using an ultra-micro-spectrophotometer. The formula for computation is as follows: Number of copies (copies/μL) = (6.022 × 10^23^/mole) ×plasmid concentration (ng/μL)/(plasmid length × 660 × 10^9^). The PRRSV standard plasmid was diluted with nuclease-free water to 10^−1^, 10^−2^, 10^−3^, 10^−4^, 10^−5^, 10^−6^, and 10^−7^. Amplification curves and melting curves of standard-quality particles were obtained according to RT-qPCR, and the standard curve equation for PRRSV N protein standard quality was obtained for subsequent detection.

### 2.8. Time-of-Addition Experiment

In order to determine whether stage of the PRRSV life cycle was impacted by SP-1, MARC-145 cells were pre-, co-, or post-treated with SP-1 before PRRSV inoculation. The control group was disposed with MEM, and the supernatant was replaced with 2% MEM at 2 h post incubation. Both the PRRSV and ribavirin groups had a 2 h PRRSV infection and were then cultured with 2% MEM either containing or not containing 100 μg/mL ribavirin. The cells in the pre-treated group were treated with 100 μg/mL SP-1 for 2 h, then infected with PRRSV for 2 h, and finally cultured with 2% MEM. The cells in the co-treated group were co-treated with PRRSV and SP-1 for 2 h and then cultured with 2% MEM. The cells in the post-treated group were grown in 2% MEM containing 100 μg/mL SP-1 after being infected with PRRSV for two hours. The cells were gathered to assess the expression level of the PRRSV N gene at 24, 36, and 48 h post treatment.

### 2.9. Virus Assay

#### 2.9.1. Virus Adsorption Assay

Cells were added to 6-well plates at a density of 2 × 10^5^/mL with 2% MEM at 2 mL per well. The control group, PRRSV group, ribavirin group, and SP-1 treatment (25, 50, and 100 μg/mL) groups were set with three replicates. The cells were first pre-chilled at 4 °C for 1 h, then cultured with 2% MEM, 2% MEM containing PRRSV, a mixture of PRRSV and 2% MEM containing 100μg/mL ribavirin, a mixture of PRRSV and 2% MEM containing SP-1 (25, 50, and 100 μg/mL) at 4 °C for another 1 h in an incubator, respectively. The cells were harvested for detecting the expression level of the PRRSV N gene using RT-qPCR.

#### 2.9.2. Virus Replication Assay

The control group, PRRSV infection group, ribavirin group (100 μg/mL), and SP-1 (25, 50, and 100 μg/mL) treatment group were set. After infection with PRRSV for 2 h and washing thrice with PBS, MARC-145 cells were cultured with 2% MEM containing ribavirin or SP-1 for 24 h. The cells were collected to detect PRRSV N gene levels using RT-qPCR.

#### 2.9.3. Virus Release Assay

The cells were added to 12-well plates. Each well was 1.0 mL with a concentration of 1 × 10^6^ cells/mL. The cells in the PRRSV infection group, ribavirin group, and SP-1 treatment groups were infected with PRRSV, while the cells in the control group were disposed with MEM. After 2 h infection, the cells were cultured with 2% MEM, 2% MEM containing ribavirin at 100 μg/mL, and 2% MEM containing SP-1 at 25, 50, and 100 μg/mL, respectively. The supernatant was collected 48 h post treatment to measure the viral DNA copy numbers and the virus titer.

### 2.10. Effect of SP-1 on CD163 Receptor Expression in PRRSV-Infected Cells

The MARC-145 cell density was adjusted to 2 × 10^5^/mL with 2% MEM and added to 6-well plates at 2000 μL/well. The cells in the PRRSV infection group, ribavirin group, and SP-1 treatment groups were infected with PRRSV for 2 h, while the cells in the control group were disposed with MEM. Then, the supernatant was replaced with 2%-MEM, 2%-MEM containing ribavirin at 100 μg/mL, and 2%-MEM containing SP-1 at 25, 50, and 100 μg/mL, respectively. The cells were collected 48 h post treatment to measure the mRNA expression of the CD163 receptor.

### 2.11. Detection of Inflammatory and Antioxidant Indexes

The control group, PRRSV infection group, and SP-1 treatment groups were respectively set with 3 replicates. The cells in the PRRSV infection group and SP-1 treatment groups were infected with PRRSV for 2 h at 37 °C, while the cells in the control group were disposed with MEM. Then, the cells were treated with 2%-MEM or 2%-MEM containing SP-1 (25, 50, and 100 μg/mL). The cells were collected at 24 h to measure the mRNA expression of Nrf2, TNF-α, HO-1, and TXNIP or detect the protein expression levels of NQO1, HO-1, and p-p65.

### 2.12. RT-qPCR Assay

The primers listed in Table 1 were used for PCR amplification. Gene expression was based on the level of GAPDH mRNA, and fold change analysis was conducted using the 2^−ΔΔCT^ comparative threshold method.

### 2.13. Western Blot Analysis

The total protein was collected using RIPA lysis buffer. Protein (30μg) were transferred onto PVDF membranes after separation via SDS-PAGE. Then, after PVDF membrane blocking with 5% skim milk at 4 °C, overnight, the membranes were incubated with monoclonal antibodies of phospho-NF-κB p65, HO-1, NQO1, and β-actin at a dilution of 1:2000 in 5% skim milk. After washing thrice with TBST for 10 min each time, the membranes were incubated for 1 h with secondary HRP-conjugated antibodies at 37 °C. Chemiluminescence was performed using chemiluminescent HRT substrate and visualized using an Image Quant LAS 500 System (General Electric Company, Boston, MA, USA).

### 2.14. Immunofluorescence Assays

The control group, PRRSV infection group, and SP-1 treatment group (100 μg/mL) were set. MARC-145 cells were disposed with MEM for 2 h, and then cultured with 2% MEM in the control group. Cells in the PRRSV infection group and SP-1 treatment group were infected with PRRSV for 2 h, then cultured with 2% MEM or 2% MEM containing 100 μg/mL SP-1 for 24 h. Next, 0.1% Triton X-100 was used to permeabilize treatment after cells were fixed with 4% paraformaldehyde at 4 °C. An overnight incubation with PRRS virus nucleocapsid protein mAb followed, and then the cells were treated for 1 h with Cy3-labeled Goat Anti-Rabbit IgG (H + L). The cells were then seen using an EVOS M5000 Imaging System after being counterstained with 1 μg/mL DAPI staining solution for 15 min.

### 2.15. Statistical Analyses 

One-way analysis of variance (ANOVA) was used to obtain the results of all statistical studies using SPSS 23.0. The results are represented as mean ± S.D., and the significant differences (*p* < 0.05) or extremely significant differences (*p* < 0.01) are designated with * or ** in the figures.

## 3. Results and Discussion

### 3.1. Chemical Analysis and Microstructure of SP-1

**Figure 1:**
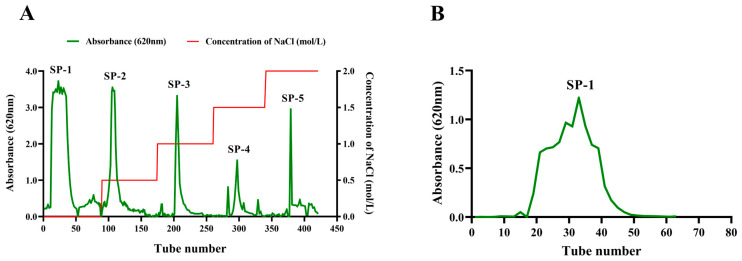
(**A**) Elution curve of *Sargassum* polysaccharide on DEAE–Sepharose Fast Flow (2.6 × 60 cm). The horizontal coordinate represents the number of elution tubes, and the vertical coordinate represents the absorbance value (620 nm) of polysaccharide determination. SP-1: Tube 0–89; SP-2: Tube 90–174; SP-3: Tube 175–260; SP-4: Tube 261–339; SP-5: Tube 340–421. (**B**) Elution curve of SP-1 on Sephacryl S200HR column (1.6 × 100 cm).

The crude *Sargassum* polysaccharide was obtained with a yield of 17.13% after the optimization of the extraction process of *Sargassum* polysaccharide via enzymatic hydrolysis and alcoholic precipitation. The crude *Sargassum* polysaccharide was separated using DEAE-Sepharose fast-flow eluting with deionized water and NaCl solution in concentrations of 0.5, 1.0, 1.5, and 2.0 mol/L. Five fractions were obtained, including SP-1, SP-2, SP-3, SP-4, and SP-5 (Figure 1A). The SP-1 component had the highest yield, reaching 52.30%. The SP-1 sample was individually eluted with Sephacryl S200HR to obtain one purified component SP-1. The SP-1 elution curve exhibited one approximate symmetrical peak, indicating that SP-1 was a homogeneous heteropolysaccharide (Figure 1B). Using the anthrone–sulfuric acid method, the polysaccharide content of SP-1 was found to be 93.2%.

### 3.2. UV–Vis Spectra

**Figure 2:**
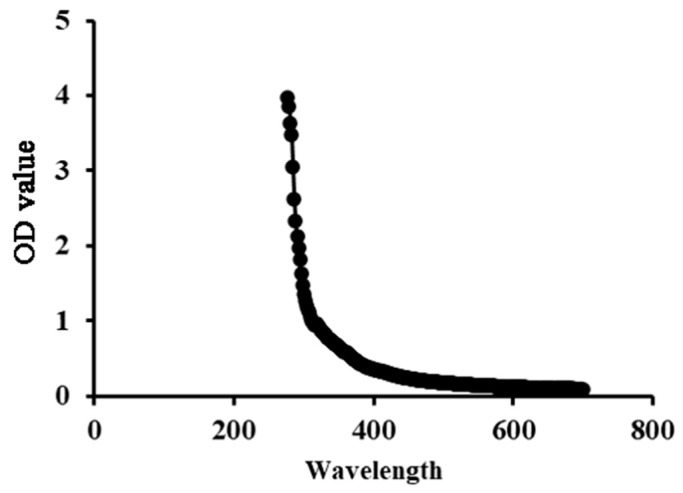
UV spectrum of SP-1 in the 200~700 nm range.

The low absorption levels at 260~280 nm in the UV–Vis spectra indicated the low nucleic acid and protein content of SP-1, indicating its high purity (Figure 2).

### 3.3. Determination of Molecular Weight (MW) and Monosaccharide Composition

**Figure 3:**
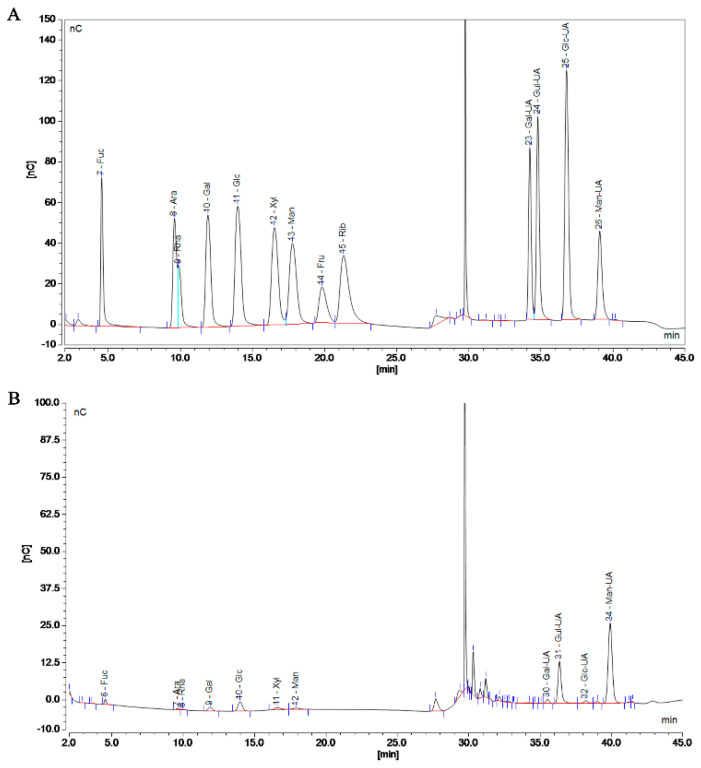
Chromatogram of standard (**A**) and SP-1 (**B**). (**A**) Left to right: Fucose (Fuc), Arabinose (Ara), Rhamnose (Rha), Galactose (Gal), Glucose (Glc), Xylose (Xyl), Mannose (Man), Fructose (Fru), Ribose (Rib), Solvent, Galacturonic acid (Gal−UA), Guluronic acid (Gul−UA), Glucuronic acid (Glc−UA), and Mannuronic acid (Man−UA). (**B**) Left to right: Fucose (Fuc), Arabinose (Ara), Rhamnose (Rha), Galactose (Gal), Glucose (Glc), Xylose (Xyl), Mannose (Man), Solvent, Galacturonic acid (Gal−UA), Guluronic acid (Gul−UA), Glucuronic acid (Glc−UA), and Mannuronic acid (Man−UA).

Polymers are mixed systems of different-molecular-weight homologues, so the molecular weight of a polymer is an average value. The MW of SP-1 was determined to be 290.37 kDa using gel chromatography, laser light scattering, and differential detection (Figure S2). The monosaccharide composition of SP-1 is shown in Figure 3A,B and specified in Table S1. Compared to the standard, SP-1 exhibited the highest content of mannuronic acid.

In this study, the monosaccharide composition of SP-1 was mainly mannuronic acid, guluronic acid, glucose, fucose, and galactose, and the MW of SP-1 was determined to be 290.37 kDa. Natural D-mannuronic acid is a constituent monosaccharide of alginate in brown algae, which is consistent with the high content of β-D-mannuronic acid in *Sargassum* spp. Generally, there is a positive correlation between alduronic acid content and antioxidant activity. Mannuronic acid has been shown to have a good inhibitory capacity against hydroxyl radicals and to scavenge superoxide anion radicals [19], which may be an important reason for the antioxidant capacity of SP-1. Cao [20] obtained top-phase *Sargassum* polysaccharide (SPP-1) and aqueous-phase *Sargassum* polysaccharide (SPP-2) through microwave-assisted aqueous two-phase extraction. The MWs of SPP-1 and SPP-2 were 1518.6 kDa and 50.6 kDa, respectively. In Li’s experiment, the DEAE-Sepharose fast-flow column was used to retrieve the *Sargassum* polysaccharide (SP-P1)’s water component, which was made up of two polysaccharides with average MWs of 323.6 and 10.7 kDa [21]. Owing to the differences in *Sargassum* species, extraction methods, and polarities of polysaccharides, the monosaccharide compositions of *Sargassum* polysaccharide show a significant difference, so the polysaccharides present different MWs due to their different proportions.

### 3.4. FT-IR Analysis

**Figure 4:**
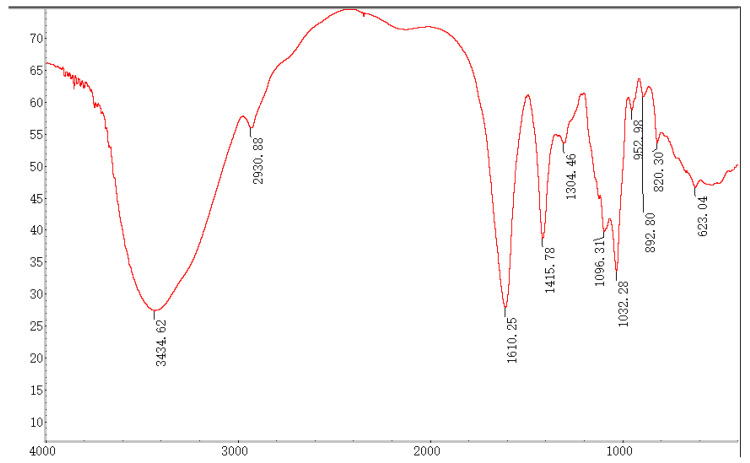
FT-IR spectrum of SP-1.

In the FT-IR spectrum, the absorption bands at 3600~3200 cm^−1^ were assigned to the typical stretch vibration of –OH, and the broad bands in this region represent the characteristic peak of polysaccharides. The signal at 3434.62 cm^−1^ represents the telescopic vibration absorption peak of O–H, and the broadband at 2930.88 cm^−1^ is attributed to the C–H telescopic vibration [20]. The signals at 1610.25 and 1096.31 cm^−1^ indicated the typical stretching vibrations of COO- and C–O were contained in SP-1 [22,23]. Furthermore, the respective characteristic absorption peaks at 892.80 and 820.30 cm^−1^ suggested the presence of α- and β-glycosidic bonds [24] in SP-1 (Figure 4).

### 3.5. Methylation Analysis

**Figure 5:**
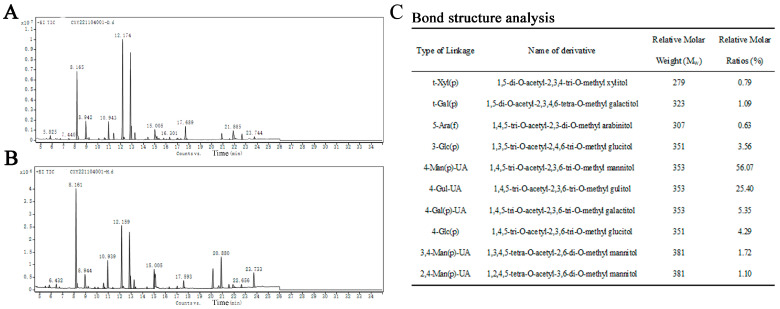
(**A**) Spectrum of total ion flow of SP-1 (D generation). (**B**) Spectrum of total ion flow of SP-1 (H generation). (**C**) Main glycosidic bond of SP-1.

The structural details of SP-1 (Figure 5A,B) were investigated through the application of methylation and GC-MS techniques to analyze the glycosidic bond types. The relative molar ratio of different glycosidic linkages in the sample was determined through dividing the chromatographic peak area ratio by the molecular weight of the corresponding derivative. The main glycosidic bond types of SP-1 are specified in Figure 5C.

### 3.6. Structure Prediction of SP-1 via NMR Spectra

**Figure 6:**
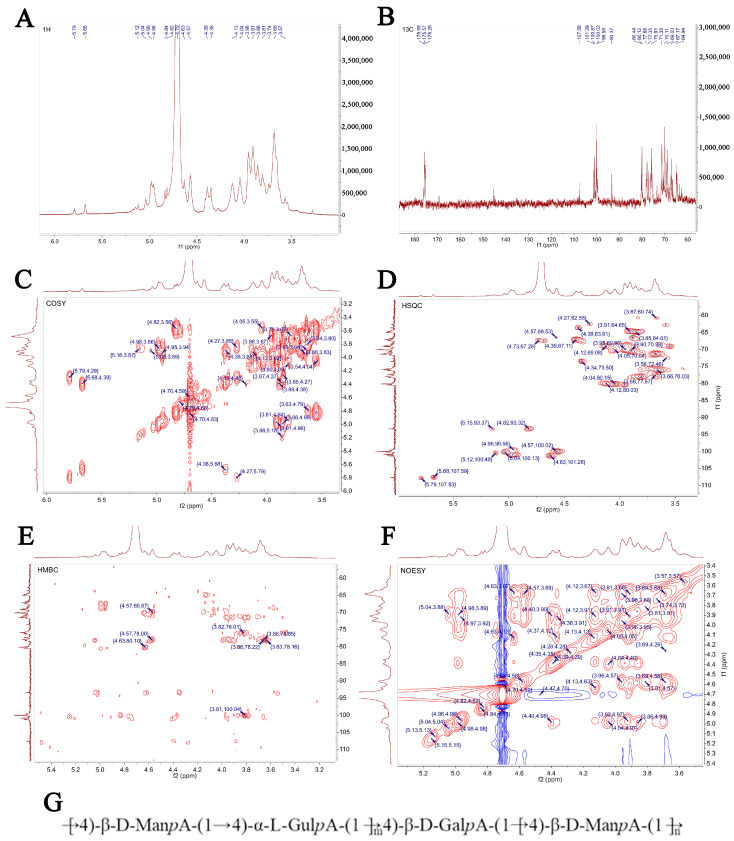
NMR spectrum and structure of SP-1. (**A**) ^1^H NMR spectrum; (**B**) ^13^C NMR spectrum; (**C**) HH-COSY spectrum; (**D**) HSQC spectrum; (**E**) HMBC spectrum; (**F**) NOESY spectrum; (**G**) bonding structures of SP-1.

The signal of SP-1 in ^1^H NMR was concentrated at δ 3~6 ppm. The heterocephalic hydrogen signals of δ 4.5~4.8 ppm and δ 4.8~5.8 ppm (Figure 6A) indicated that SP-1 contains β-glycosidical and α-glycosidical configurations [25]. Multiple coupled signal peaks were identified in the δ 4.5~5.8 ppm heterocephalic signaling region, indicating that this sample contains a variety of glycosyl residues, and the chemical shifts corresponding to heterocephalic hydrogen were δ 4.57, 4.98, 4.63, 4.82, 5.04, 5.12, and 5.68 ppm, respectively, represented by residues A, B, C, D, E, F, and G. The non-heterocephalic hydrogen signal was mainly concentrated in the δ 3.2~4.2 ppm region, and the strong signal peak around δ 4.71 ppm was the solvent peak.

The anomeric carbon signal of polysaccharides in ^13^C NMR was concentrated at 95~110 ppm. Figure 6B shows the presence of both α (δ 95~101 ppm) and β (δ 101~105 ppm) configurations, as evidenced by the anomeric carbons observed in the signal range of 93.37 to 107.58 ppm. This observation aligns with the findings of the ^1^H NMR spectrum. Multiple signal peaks were identified in the heterocephalic region during the detection of SP-1 samples, and combined with the crossover peaks of the ^13^C NMR spectrum and the HSQC–HMBC spectral heterocephalic region (Figure 6D,E), the heterocephalic signals of residues A, B, C, D, E, F, and G were determined to be δ 4.57/100.02, 4.98/100.87, 4.63/101.28, 4.82/93.32, 5.04/100.13, 5.12/100.49, and 5.68/107.59 ppm. Combined with sample bonding structure (methylation) information, anomeric carbon signaling, and literature reports, it was speculated that glycosyl residue A was →6)-β-D-ManpA- (1→, residue B was →4)-α-L-gulpA-(1→, residue C was →4)-β-D-GalpA-(1→, and its chemical shifts of ^1^H and ^13^C were attributed, and the results are shown in Table 2. Due to the low content of glycosyl residues D, E, F, and G, the signal in the NMR pattern was weak, and it was difficult to infer its residue information. The NMR analysis (Figure 6A–F) revealed that the polysaccharide primarily consists of a linear chain composed of →4)-β-D-ManpA-(1→, →4)-α-L-GulpA-(1→, and a minor amount of →4)-β-D-GalpA-(1→. Figure 6G illustrates the proposed structure of the polysaccharide chain.

### 3.7. SEM Analysis and XRD Analysis

**Figure 7:**
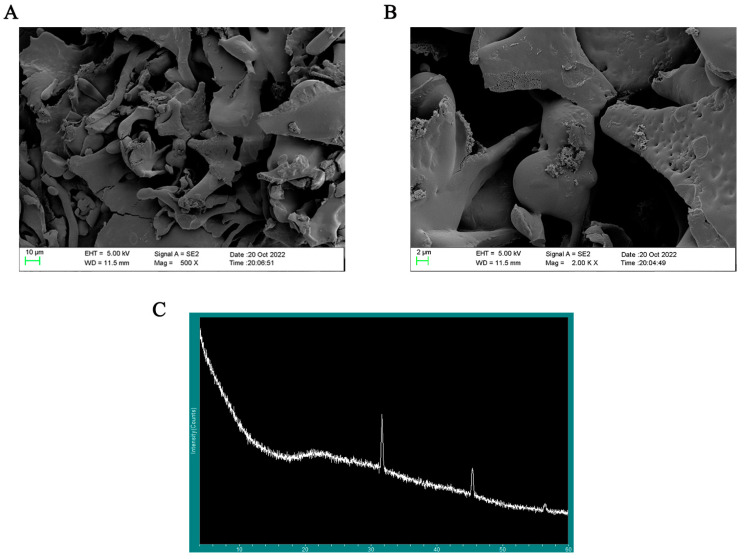
(**A**) Typical SEM images of SP-1 at magnification of 500×. (**B**) Typical SEM images of SP-1 at magnification of 2000×. (**C**) X-ray diffraction detection of SP-1.

SP-1 showed up as an amorphous lamellar structure at a magnification of 500×, as seen in Figure 7A. At a magnification of 2000×, the smooth surface of SP-1 (Figure 7B) revealed the presence of fissures and holes, potentially attributed to van der Waals forces and intra- and intermolecular hydrogen bonding [18,26]. These observations align with the findings reported by Yuan et al. [26].

Most polysaccharides have an amorphous or semi-crystalline structure. The extracted polysaccharides are water-soluble branched-chain biopolymers [27]. Therefore, it is foreseen that the extracted polysaccharides will be highly amorphous polymers or compounds with a small amount of crystalline structure. In general, polysaccharides as macromolecules do not suddenly show a very narrow range of diffraction peaks in a section of a flat region. Figure 7C reveals rounded broad peaks on the whole in SP-1, indicating the presence of an amorphous structure within the range of 5 to 80 degrees [28]. Combined with polysaccharide content levels, two reflections at 32 and 45 degrees indicated the presence of diffraction peaks at this site could be due to trace inorganic salt impurities with no practical significance.

### 3.8. Cytotoxic Effects of SP-1 on MARC-145 Cells

**Figure 8:**
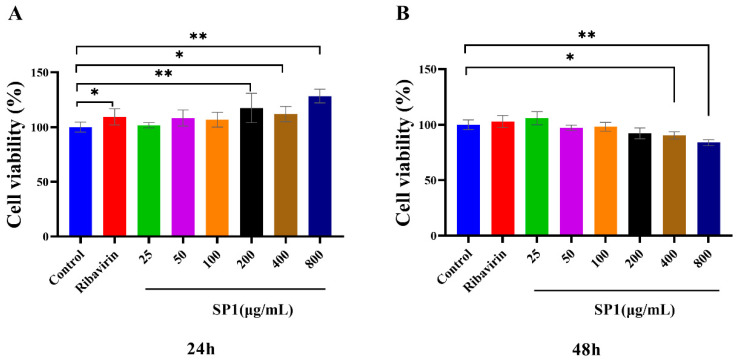
Cytotoxic effects of SP-1 in MARC-145 cells after (**A**) 24 h and (**B**) 48 h. Notes: Compared with the control group, “*” indicates a significant difference (*p* < 0.05), and “**” indicates an extremely significant difference (*p* < 0.01).

The cytotoxic effects of SP-1 on MARC-145 cells were determined using the CCK-8 kit at 24 and 48 h post treatment (Figure 8A,B). The results revealed no cytotoxic effects of SP-1 at a dosage from 25 to 800 μg/mL at 24 h. At 48 h, there was no insignificant cytotoxic effects of SP-1 from 25 to 200 μg/mL. SP-1 treatment at 400 or 800 μg/mL could down-regulate cell viability significantly (*p* < 0.05, *p* < 0.01), indicating that SP-1 concentrations from 25 to 100 μg/mL were safe on MARC-145 cells within 48 h. Thus, SP-1 concentrations at 25, 50, and 100 μg/mL were selected for the following experiments.

### 3.9. Construction and Identification of Recombinant Plasmids of N Gene

The nucleocapsid N gene recombinant plasmid was detected with PCR, and a specific amplification band at 173 bp was observed, indicating that the nucleocapsid N gene recombinant plasmid was successfully constructed (Figure S3). The recombinant-positive plasmids were analyzed through comparing them with NCBI and exhibited 100% homology with the expected gene fragment nucleic acids. The standard curve of PRRSV copy number and CT value (*Y* = −0.3136 *X* + 11.224, R^2^ = 0.9969) was obtained through diluting the recombinant plasmid for a RT-qPCR reaction (melting temperature (TM) = 86 °C), showing a single specific peak, indicating that the established RT-qPCR method demonstrated good specificity.

### 3.10. SP-1 Inhibits Virus Adsorption and Internalization

**Figure 9:**
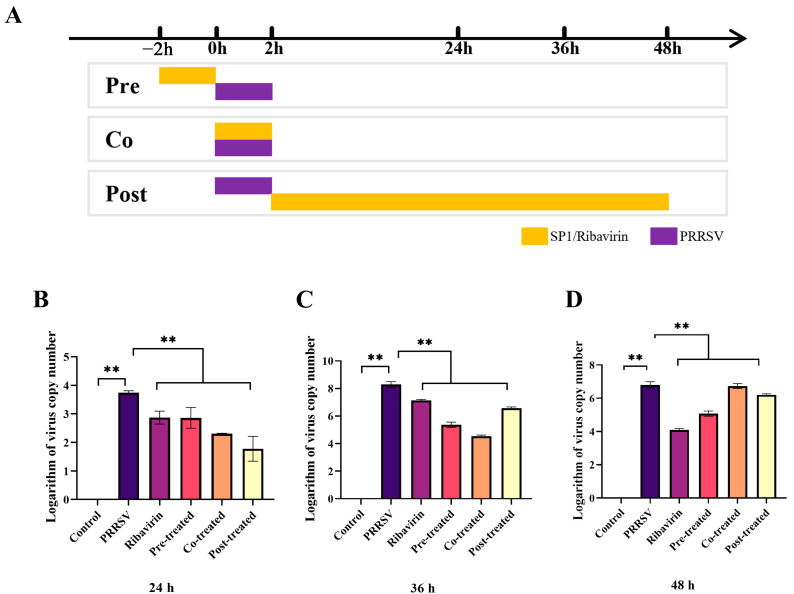
Screening procedure time course (**A**) and PRRSV replicated levels at 24 h (**B**), 36 h (**C**), 48 h (**D**). Notes: “**” indicates an extremely significant difference (*p* < 0.01).

As evident in Figure 9B–D, the results demonstrated that pre-, co- or post-treatment with SP-1 significantly suppressed PRRSV replication at 24, 36, and 48 h, respectively (*p* < 0.01).

**Figure 10:**
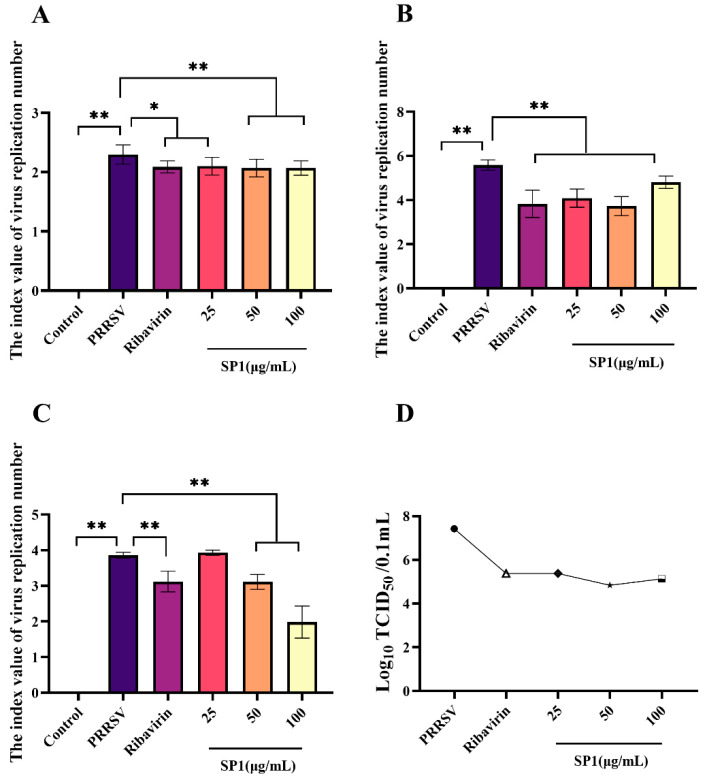
(**A**) Adsorption assay. (**B**) Replication assay. (**C**) Release assay. (**D**) Progeny virus titer detection in cell supernatant, “●” PRRSV group, “△” Ribavirin group, “◆” SP1 25 ug/mL group, “★” SP1 50 ug/mL group, “□” SP1 100 ug/mL group. Notes: “*” indicates a significant difference (*p* < 0.05), and “**” indicates an extremely significant difference (*p* < 0.01).

SP-1 appeared to intervene with PRRSV at the adsorption stage as evidenced by the dose-dependent reduction in virus replication following SP-1 therapy (Figure 10A, *p <* 0.05, *p <* 0.01). Additionally, PRRSV replication was strongly suppressed by SP-1 treatment at 25, 50, or 100 μg/mL. And in the virus replication experiment, the amount of PRRSV replicating in MARC-145 cells fell dramatically (Figure 10B). To investigate the role of SP-1 in PRRSV release, the viral DNA copy number and virus titer of PRRSV were measured in the supernatant of MARC-145 cell culture at 48 h (Figure 10C). Treatment with SP-1 (Figure 10D) resulted in a significant down-regulation of both N gene copy numbers and virus titer.

Previous research has demonstrated that marine polysaccharides possess diverse antiviral mechanisms. These mechanisms include direct virucidal action, the inhibition of viral adsorption, the inhibition of virus internalization and uncoating, the suppression of viral transcription and replication, and the enhancement of host antiviral immune responses [29]. λ-type carrageenan could inhibit herpes simplex virus replication through binding HSV tightly to result in the inactivation of HSV virions [30]. The inhibition of Newcastle disease virus infection can be achieved through the suppression of virus adsorption using sulfated Chuanmingshen violaceum polysaccharides [31]. The inhibitory effect of fucoidan KW on viral neuraminidase activity resulted in the prevention of IAV release, while also interfering with endocytosis and EGFR internalization through the regulation of the NF-κB and AKT pathways, ultimately providing protection against IAV infection [32]. The finds showed that SP-1 had effective antiviral activity during the viral adsorption, replication, and release processes. Notably, the antiviral effect of SP-1 was comparable to, or even superior to, that of the ribavirin group. These results demonstrate the potential of SP-1 as a preventive and therapeutic measure against PRRSV infection.

### 3.11. SP-1 Down-Regulated the Expression Level of Key CD163 Receptor, Inhibiting Viral Adsorption and Internalization

**Figure 11:**
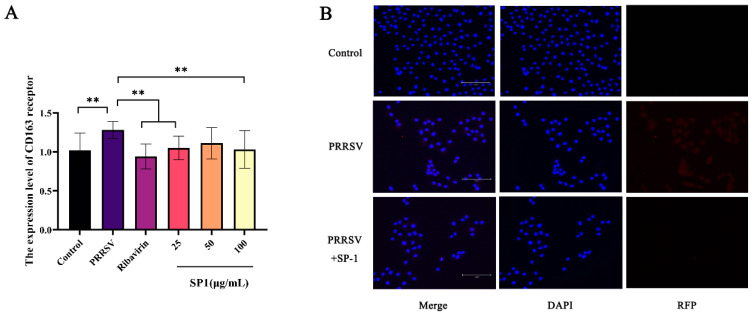
(**A**) The expression level of CD163 receptor. (**B**) Immunofluorescence detection in PRRSV N protein. Notes: “**” indicates an extremely significant difference (*p* < 0.01).

CD163 is the core receptor involved in PRRSV infection, mainly playing a role in the viral capsid and genomic release [33]. The RT-qPCR results (Figure 11A) showed a considerable up-regulation of the CD163 receptor expression level in the PRRSV-infected group (*p <* 0.01). After 24 h treatments, SP-1 at 25 or 100 μg/mL and ribavirin at 100 μg/mL down-regulated the CD163 expression level (*p <* 0.01). The cells were probed for PRRSV N protein and observed under a fluorescence microscope (Figure 11B). The results revealed that PRRSV could enter into cells 24 h post infection. After SP-1 treatment for 24 h, the fluorescence of N protein in the cytoplasm was significantly weakened, indicating that SP-1 could inhibit PRRSV viral infection at the stages of adsorption and internalization.

Numerous studies have demonstrated that downregulating CD163 receptor expression hampers PRRSV invasion and internalization. Previous studies have shown that CD163-knockout pigs exhibit strong resistance to PRRSV, suggesting that PRRSV infection relies on the expression of the CD163 receptor [34,35]. The activation of the NF-κB pathway could be inhibited and PRRSV infection could be weakened through blocking scavenger receptor cysteine-rich domain 5–9 in the CD163 receptor before or after virus attachment [36]. SP-1 significantly decreased CD163 receptor expression levels, indicating that SP-1 could inhibit PRRSV infection through resisting PRRSV invasion and internalization.

### 3.12. SP-1 Up-Regulated the Antioxidant Pathway, Inhibiting PRRSV-Induced Inflammation 

**Figure 12:**
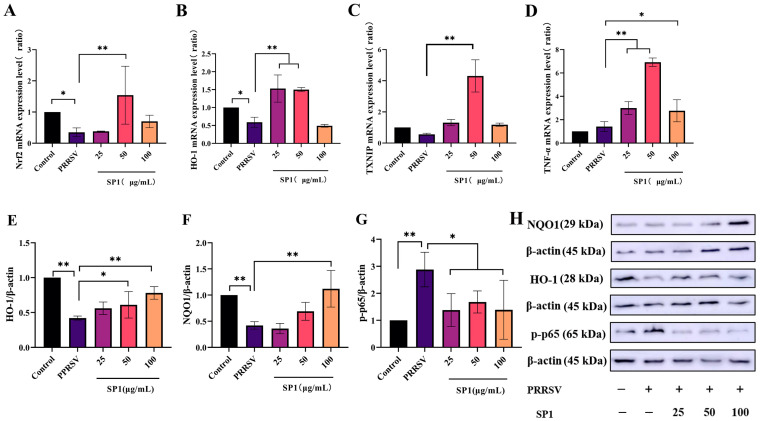
mRNA expression level of Nrf2 (**A**), HO-1 (**B**), TNF-α (**C**), and TXNIP (**D**); protein expression level of HO-1 (**E**), NQO1 (**F**), and p-p65 (**G**); protein band (**H**). Notes: (**A**–**G**): “*” indicates a significant difference (*p* <0.05), and “**” indicates an extremely significant difference (*p* < 0.01). (**H**): “−” means not included, “+” means included.

Figure 12A–D illustrate a significant decrease in Nrf2 and HO-1 mRNA expression levels 24 h after infection (*p <* 0.05). After 24 h treatment with SP-1, SP-1 at 50 μg/mL up-regulated Nrf2 and TXNIP mRNA expression levels in PRRSV-infected cells (*p <* 0.05). Additionally, the expression levels of HO-1 and TNF-α were significantly increased for 24 h after treatment with SP-1 at 25 or 50 μg/mL (*p <* 0.01). In brief, SP-1 alleviated cell injury responses through up-regulating the expression of antioxidant genes (Nrf2, HO-1, and TXNIP) in PRRSV-infected cells. Meanwhile, SP-1 raised TNF-α levels to increase immune responses in the cells.

As evident in Figure 12E–H, the protein expression levels of HO-1, NQO1, and p-p65 were detected to verify the role of SP-1 in the antioxidant pathway. According to the findings, SP-1 treatment at 100 μg/mL significantly increased the NQO1 protein level (*p* < 0.01), and SP-1 at 50 or 100 μg/mL significantly up-regulated the protein level of HO-1 (*p* < 0.05 or *p* < 0.01), indicating that SP-1 could inhibit PRRSV infection through regulating the Nrf2-HO1 pathway. In addition, the p-p65 protein level exhibited significant up-regulation after 24 h (*p <* 0.01). SP-1 treatment at 25, 50, or 100 μg/mL significantly decreased the level of p-p65 protein (*p <* 0.05).

Nrf2 is a key regulator of cellular antioxidant stress mechanisms, regulates the transcription of glutathione (GSH) and thioredoxin (TXN) antioxidant system components, and is closely related to the expression of HO-1 [37]. Nrf2 could protect organs against virus-induced oxidative stress injury [38]. Many studies have shown that viral infections inhibit the activation of the Nrf2-HO-1 pathway, which induces oxidative stress and inflammatory responses. Enterovirus 71 (EV71) infection could reduce the number of peroxisomes, enhance ROS production, and inhibit the activation of the Nrf2/HO-1 pathway, thereby inducing apoptosis and autophagy in neural cells [39]. In the normal state of a cell, TXNIP is located in the nucleus, and when ROS accumulation in the cell increases, it induces TXNIP to shuttle into the cytoplasm or mitochondria, inhibiting the antioxidant capacity of Trx through binding to Trx [40]. The TXNIP transcript and protein levels presented a higher level in human acute myeloid leukemia (AML) blasts [41]. Hepatitis B virus X protein enhanced the transcription and replication of HBV and increased the expression level of TXNIP, thereby mediating the metastasis of hepatocellular carcinoma [42]. *Sargassum* polysaccharide belongs to the brown algae polysaccharide sulfate, which has a unique sulfate and can improve antioxidant activity. Previous studies have demonstrated that *Sargassum* polysaccharide can prevent H_2_O_2_-induced RAW264.7 cell oxidative stress through boosting intracellular SOD and glutathione peroxidase (GPx) activities and decreasing intracellular ROS and NO levels [43]. The expression level of TXNIP was down-regulated after PRRSV infection. It may be related to the inhibition of TXNIP binding to Trx to increase the antioxidant level of cells. Our study revealed that SP-1 exerted anti-PRRSV activity through upregulating the mRNA expression levels of Nrf2, HO-1, and TXNIP in MARC-145 cells and stimulating Nrf2-mediated antioxidant pathways. The results showed that although both the protein expression level and mRNA expression level of HO-1 were up-regulated by the regulatory effects of the drugs over the same duration of action, there were some differences in the changes induced by SP-1. It may be due to the lagging expression of the protein. Additionally, SP-1 counteracts virus-induced inflammation through resisting the phosphorylation of NF-κB p65. These findings may offer a helpful therapeutic strategy for PRRS management.

## 4. Conclusions

In this study, the water-soluble component of *Sargassum* polysaccharide (SP-1) was isolated and purified from *Sargassum* on Weizhou Island. SP-1 is a novel polysaccharide which is mainly composed of →4)-β-D-ManpA-(1→, →4)-α-L-GulpA-(1→, and a small amount of→4)-β-D-GalpA-(1→. SP-1 can resist PRRSV infection through inhibiting virus adsorption, replication, and release, and SP-1 treatment could enhance antioxidant gene (Nrf2, HO-1, TXNIP) and protein expression (HO-1, NQO1) to enhance antiviral ability in MARC-145 cells. In summary, SP-1 may be a potential agent against PRRSV infection. It plays an anti-PRRSV role mainly through inhibiting PRRSV at the stages of adsorption, replication, and release, enhancing the Nrf2-HO1 antioxidant pathway.

## Figures and Tables

**Table 1:** Nucleotide sequences of specific primers.

Target Gene	Accession	Primer Sequence	PCR Production (bp)
GAPDH	103218453	5′-TCATGACCACAGTCCATGCC-3′	144
		5′-GGATGACCTTGCCCACAGCC-3′	
CD163	103218507	5′-AAGCCACAACAGGTCAGTCATTCC-3′	117
		5′-CTGTAAGCCGCTGTCTCTGTCTTC-3′	
Nrf2	103217393	5′-ATTCAATGATTCTGACTCTG-3′	199
		5′-CGTATCCCCAGAAGAATGTA-3′	
HO-1	103223230	5′-CTTCAAGCTGGTGATGGC-3′	219
		5′-TGGAGCCGCTTCACATAG-3′	
TXNIP	103224057	5′-CGACCCTGAAAAAGGTGTAC-3′	261
		5′-CGAACTTGTACTCATATTTG-3′	
TNF-α	119626817	5′-TCCTCAGCCTCTTCTCCTTCCT-3′	70
		5′-ACTCCAAAGTGCAGCAGACAGA-3′	

**Table 2:** Chemical shift assignment of glycosidic linkages.

Glycosyl Residues	Residue	Chemical Shifts (ppm)					
		H1/C1	H2/C2	H3/C3	H4/C4	H5/C5	H6/C6
→4)-β-D-ManpA-(1→	A	4.57	3.96	4.12	3.67	3.68	/
		100.02	70.04	69.08	75.98	71.35	175.57
→4)-α-L-GulpA-(1→	B	4.98	3.85	4.05	3.81	4.39	/
		100.87	64.78	70.04	77.85	67.13	175.29
→4)-β-D-GalpA-(1→	C	4.63	3.93	3.91	3.96	4.42	/
		101.28	69.16	70.24	80.42	67.11	175.99

## Data Availability

All data generated or analyzed during this study are included in this published article (and its Supplementary Information Files).

## Supplementary Materials

The following supporting information be downloaded at: https://www.mdpi.com/article/10.3390/antiox12101832/s1, Table S1: Monosaccharide composition; Figure S1: The process of extraction, isolation and purification of *Sargassum weizhouense*; Figure S2. Plot of absolute molecular weight analysis; Figure S3: Construction of plasmid and establishment of qRT-PCR standard curve.

## Abbreviations

AML: acute myeloid leukemia; CD163: scavenger receptor cysteine-rich type 1 protein M130; D_2_O: deuterium oxide; EI: electron bombardment ion source; EV71: enterovirus 71; GSH: glutathione; HO-1: heme oxygenase 1; NF-κB: nuclear factor kappa-B; NMR: nuclear magnetic resonance; N protein: nucleocapsid protein; NQO1: NAD(P)H dehydrogenase: quinone 1; Nrf2: nuclear factor erythroid2-related factor 2; PCV2: porcine circovirus type 2; p-p65: Phospho-NF-κB p65; PRRS: porcine reproductive and respiratory syndrome; PRRSV: porcine reproductive and respiratory syndrome virus; SP: Sargassum polysaccharide; SPP-1: top-phase Sargassum polysaccharide; SPP-2: aqueous-phase Sargassum polysaccharide; TNF-α: tumor necrosis factor alpha; TXN: thioredoxin; TXNIP: thioredoxin interacting protein.
