# Supplementary material for: A Novel Polysaccharide from Sargassum weizhouense: Extraction Optimization, Structural Characterization, Antiviral and Antioxidant Effects
Source: Antioxidants (Basel). 2023 Oct 6;12(10):1832. doi: 10.3390/antiox12101832 (PMC10604564; doi:10.3390/antiox12101832)
Supplement: Supplementary file 1 [file antioxidants-12-01832-s001.zip › antioxidants-2616700-SI.pdf]

Supplemental reagents

Ethanol, trichloroacetic acid, anthracene, sulfuric acid, α-naphthol, iodine, potassium iodide, indamine, glycine, and sodium chloride were all analytically pure grades. Papain, Dextran T70, Dextran T40, Dextran T10, 4% paraformaldehyde, and Dextran Blue 2000 were purchased from Solarbio Technology Co., Ltd. (Beijing, China). Minimum Eagle's medium was purchased from Sigma-Aldrich Company (Darmstadt, Germany). Fetal bovine serum (FBS) was purchased from Biological Industries (Kibbutz Beit Haemek, Israel). The Thermo Scientific™ GeneJET Viral DNA/RNA Purification kit was purchased from Thermo Fisher Scientific (Waltham, MA, USA). The EZNA® Gel Extraction Kit, PCR purification kit, and Plasmid extraction kit were purchased from OMEGA (Connecticut, USA). The pMD™18-T Vector Cloning Kit and RNAiso Plus were purchased from Takara Biomedical Technology Co., Ltd. (Beijing, China).

Table S1. Monosaccharide composition.

Name	CAS Number (Sigma)	Molecular Formula	Content (μg/mg)
Fucose (Fuc)	2438-80-4	C6H12O5	4.4807
Arabinose (Ara)	5328-37-0	C5H10O5	1.3101
Rhamnose (Rha)	10030-85-0	C6H14O6	2.1440
Galactose (Gal)	26566-61-0	C6H12O6	4.5202
Glucose (Glc)	50-99-7	C6H12O6	9.4481
Xylose (Xyl)	58-86-6	C5H10O5	3.9688
Mannose (Man)	3458-28-4	C6H14O6	3.5177
Galacturonic Acid (Gal-UA)	14982-50-4	C6H10O7	7.3167
Guluronic Acid (Gul-UA)	15769-56-9	C6H10O7	59.4252
Glucuronic Acid (Glc-UA)	6556-12-3	C6H10O7	2.6896
Mannuronic Acid (Man-UA)	6814-36-4	C6H10O7	222.3577

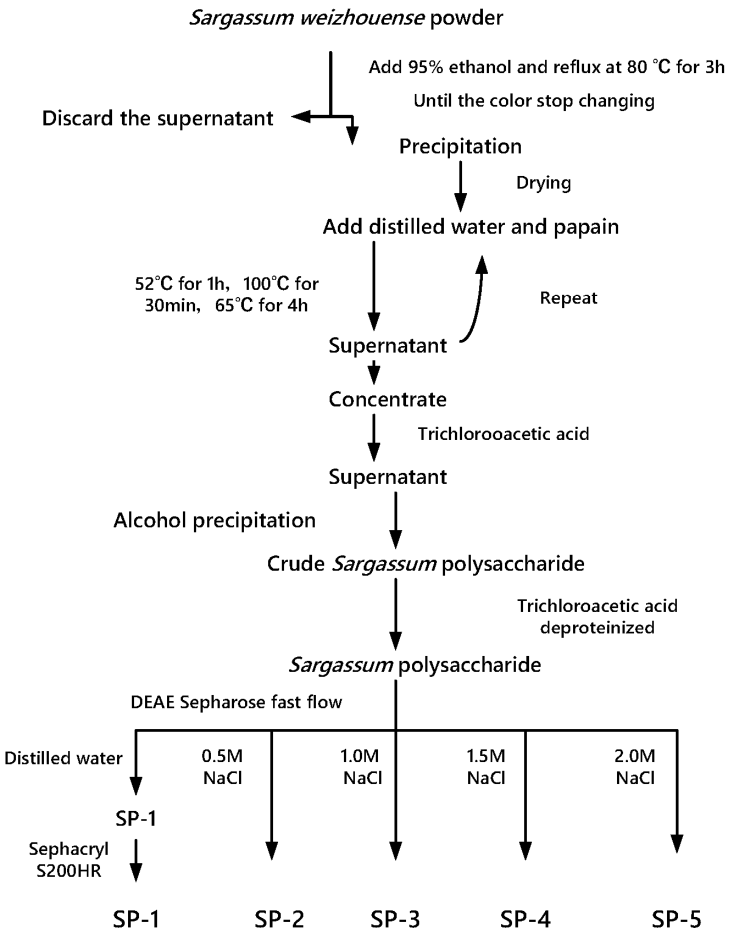

Figure S1. The process of extraction, isolation, and purification of *Sargassum weizhouense.*

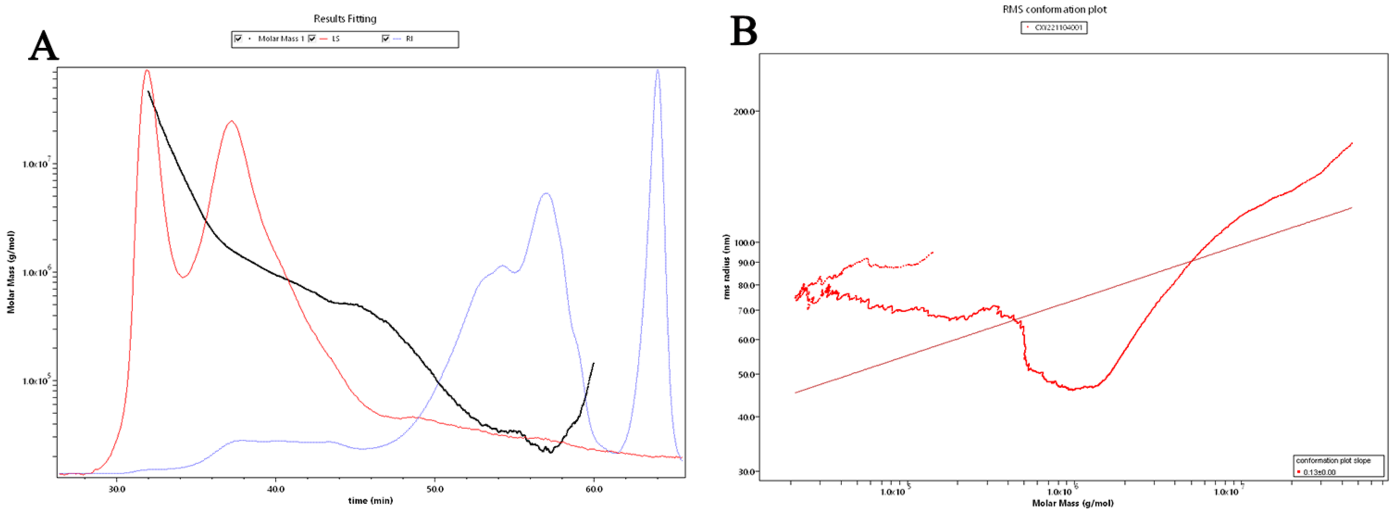

Figure S2. (**A**) Plot of absolute molecular weight analysis. Red line: the multi-angle laser light scattering signal (LS, unit: V). Blue line: the differential signal (RI, unit: RIU). Black line: the molecular weight fitted from the two signals. (**B**) The molecular configuration analysis of SP-1.

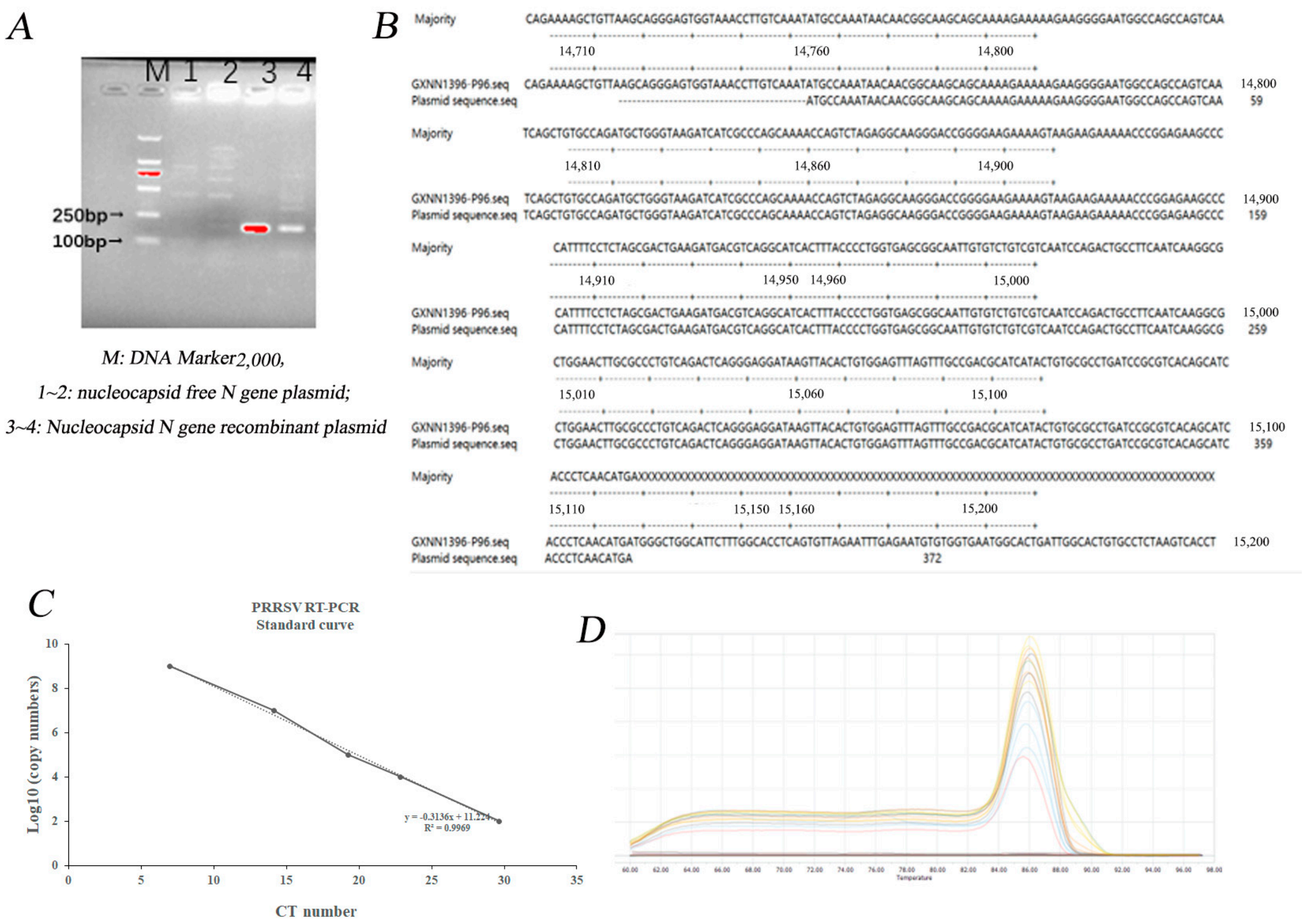

Figure S3. Construction of plasmid and establishment of qRT-PCR standard curve. (**A**) Gel electrophoresis detection of N gene recombinant plasmid. (**B**) Recombinant plasmid and PRRSV sequence alignment results. Note—the upper part is the sequence of plasmid N gene, whereas the lower part is PRRSV-GXNN1396. (**C**) Standard curve line of PRRSV qRT-PCR (CT-X). Note—The corresponding template concentrations from right to left in the above figure are: 8.396×10^9, 8.396×10^7, 8.396×10^5, 8.396×10^4, and 8.396×10^2 copies/μL. (**D**) Establishment of qRT-PCR melting curve.
